# Synthesis of amorphous trimetallic PdCuNiP nanoparticles for enhanced OER

**DOI:** 10.3389/fchem.2023.1122333

**Published:** 2023-01-30

**Authors:** Yangzi Zheng, Ruiyun Guo, Xiang Li, Tianou He, Weicong Wang, Qi Zhan, Rui Li, Ke Zhang, Shangdong Ji, Mingshang Jin

**Affiliations:** ^1^ State Key Laboratory of Multiphase Flow in Power Engineering, Frontier Institute of Science and Technology, Xi’an Jiaotong University, Xi’an, Shaanxi, China; ^2^ School of Materials Science and Engineering, Xi’an University of Science and Technology, Xi’an, Shaanxi, China; ^3^ Shaanxi Key Laboratory of Optoelectronic Functional Materials and Devices, School of Materials Science and Chemical Engineering, Xi’an Technological University, Xi’an, Shaanxi, China

**Keywords:** palladium, phosphide, amorphous, core-shell, electrocatalysis

## Abstract

Metal phosphides with multi-element components and amorphous structure represent a novel kind of electrocatalysts for promising activity and durability towards the oxygen evolution reaction (OER). In this work, a two-step strategy, including alloying and phosphating processes, is reported to synthesize trimetallic amorphous PdCuNiP phosphide nanoparticles for efficient OER under alkaline conditions. The synergistic effect between Pd, Cu, Ni, and P elements, as well as the amorphous structure of the obtained PdCuNiP phosphide nanoparticles, would boost the intrinsic catalytic activity of Pd nanoparticles towards a wide range of reactions. These obtained trimetallic amorphous PdCuNiP phosphide nanoparticles exhibit long-term stability, nearly a 20-fold increase in mass activity toward OER compared with the initial Pd nanoparticles, and 223 mV lower in overpotential at 10 mA cm^−2^. This work not only provides a reliable synthetic strategy for multi-metallic phosphide nanoparticles, but also expands the potential applications of this promising class of multi-metallic amorphous phosphides.

## 1 Introduction

The rapid consumption of fossil fuels with more and more population has caused serious social and ecological problems, including climate change, the greenhouse effect, sea level rise, and environmental pollution (Chu et al., 2012; Shih et al., 2018). Developing sustainable and clean energy conversion and storage technologies is vital to address the above-mentioned problems. Especially, electrochemical energy conversion technologies, such as water electrolysis for hydrogen production, fuel cells, and carbon dioxide conversion, have attracted numerous attention in the past few decades by virtue of the high energy conversion efficiency (He et al., 2021a; Wang et al., 2021a; Liu et al., 2021; Zhang et al., 2022; Zhou et al., 2022). Oxygen evolution reaction (OER) is evinced as one of the main rate-determining steps for clean energy production *via* electrochemical process (Li et al., 2014a; Hong et al., 2015; Xia et al., 2016). By far, the application of renewable energy conversion and storage processes is still hindered by the sluggish kinetics and low efficiency of OER originating from the four-electron process (Koper et al., 2013; Li et al., 2014b; Trotochaud et al., 2014; Chen et al., 2015; Jiao et al., 2015; Hunter et al., 2016; Reier et al., 2017; Song et al., 2018). The rational design and synthesis of electrocatalysts with high electrocatalytic activity and stability remain critical issues in the construction of high-performance electrochemical energy production systems. Extensive attempts have been made to develop advanced OER electrocatalysts to date (McCrory et al., 2013; Antolini et al., 2014; Trotochaud et al., 2014; Yu et al., 2015; Suen et al., 2017; Song et al., 2018; Zhang et al., 2018). Many types of nanomaterials, such as metal oxides, hydroxides, and layered double hydroxides (LDHs), have been reported to exhibit excellent electrocatalytic performances towards OER under alkaline conditions (McCrory et al., 2013; Trotochaud et al., 2014; Yu et al., 2015; Suen et al., 2017; Zhang et al., 2018). Nevertheless, low electrical conductivity is a crucial drawback for most oxides and hydroxides OER electrocatalysts, which may impede the ability of electron transport and cause sluggish reaction kinetics and low yields (Xu et al., 2016).

Recently, many researchers have developed doping strategies for high-efficiency electrocatalysts, which could principally modulate the electronic structure, thus enhancing the electrocatalytic activity (Xiao et al., 2022). Considering the important role of phosphorus (P) doping in improving the electrical conductivity and enhancing the intrinsic activity of metal catalysts, works related to monometallic and bimetallic phosphides, including the engineering of heterojunctions for potential TMP catalysts and phosphated bimetallic clusters on macroporous nitrogen-doped carbon, have been explored in recent years (Ryu et al., 2015; Stern et al., 2015; Liu et al., 2018a; Qin et al., 2018; Chu et al., 2019; Lv et al., 2020; Wang et al., 2021b; Guo et al., 2022). Meanwhile, according to the recently reported references, many electrocatalysts with amorphous structure have been proven to be more efficient than their crystalline counterparts (Wang et al., 2021c; Wang et al., 2022). Inspired by these, the combination of constructing an amorphous structure and P atom doping strategy is an excellent way to improve electrocatalytic performance. In recent years, it has been reported that Pd-based electrocatalysts can exhibit outstanding electrocatalytic performance for OER, especially in alkaline media (Kwon et al., 2013; Li et al., 2014a; Alegre et al., 2015). Particularly, alloying with other transition metals can effectively enhance the intrinsic catalytic activity by modulating the electronic structure of the Pd sites (Yu et al., 2016; Feng et al., 2017; Tang et al., 2017; Xu et al., 2018; Park et al., 2019; Zhu et al., 2019), thus reducing the usage of Pd catalysts. Therefore, there have been many mono- and bi-metallic phosphide nanoparticles reported in the literature previously, which can deliver superior catalytic activities relative to Pd nanoparticles. Compared with mono- and bi-metallic phosphide nanoparticles, alloying Pd with more transition metal elements can further tune the electronic structure of the Pd sites, and thus an even better catalytic performance could be expected for tri-metallic phosphide nanoparticles (Kim et al., 2018; Xu et al., 2020). However, trimetallic phosphide nanoparticles have rarely been reported, since the phase separation would be likely happened during the synthesis process due to the coexistence of trimetallic transition metal elements. Furthermore, multiple metal X-ides (including phosphides, sulfides, nitrides, and carbides) have been demonstrated increasingly to be better OER catalysts thanks to their lower free energy barrier in DFT calculations (Zheng et al., 2018; Luo et al., 2021). Therefore, the development of ternary metal phosphide catalysts for efficient electrocatalytic reactions is challenging and significant.

Herein, an effective approach has been developed for the preparation of trimetallic PdCuNiP phosphide nanoparticles with an amorphous structure based on the phosphorization treatment of Pd@PdCuNi core-shell nanoparticles. Impressively, these obtained trimetallic amorphous PdCuNiP phosphide nanoparticles exhibit long-term stability, with nearly a 20-fold increase in mass activity toward OER compared with the initial Pd nanoparticles. Moreover, the PdCuNiP nanoparticles possess an overpotential as small as 314 mV @ 10 mA cm^−2^, much smaller than that of the commercial RuO_2_ (391.5 mV) and the original Pd nanocubes (537 mV).

## 2 Materials and methods

### 2.1 Chemicals and reagents

Sodium tetrachloropalladate (Na_2_PdCl_4_, 98%), poly-(vinyl pyrrolidone) (PVP, M_w_ ≈55,000), ascorbic acid (AA, 99%), potassium bromide (KBr, 99%), CuCl_2_·2H_2_O, Ni(acac)_2_, oleylamine (OAm, 80%–90%), tri-n-octylphosphine (TOP, 90%), nafion-117 (5%), Pd/C (10 wt%) were all purchased from Sigma-Aldrich and utilized as received. KOH (85%) were purchased from Alfa Aesar. Isopropyl alcohol (C_3_H_8_O, AR) was bought from Macklin to use. Ketjen Black (ECP600JD) was purchased from Sinero. Milli-Q ultrapure water and ethanol absolute (AR, 0.79 g/mL) were used throughout all the experiments.

### 2.2 Synthesis

Synthesis of Pd nanocubes. Pd nanocubes were prepared according to the approach reported previously (Jin et al., 2011). For a typical synthesis, 11 mL of an aqueous solution containing poly-(vinylpyrrolidone) (PVP, Mw ≈55,000, 105 mg, Aldrich), L-ascorbic acid (AA, 60 mg, Aldrich), KBr (300 mg, Fisher), and sodium tetrachloropalladate (Na_2_PdCl_4_, 57 mg, Aldrich) was placed in a vial and heated at 80°C in the air under magnetic stirring for 3 h. The obtained product was collected by centrifugation and washed 4 times with water and ethanol, and then re-dispersed in 10 mL of oleylamine.

Synthesis of Pd@PdCuNi core-shell nanocrystals. Pd@PdCuNi core-shell nanocrystals were based on a modified two-step approach reported by our group previously (Li et al., 2018). (1) 4 mg of CuCl_2_·2H_2_O, 3 mL of OAm, and 1 mL of an OAm solution of Pd nanocubes were mSixed in a 50-mL round-bottomed flask and heated in an oil bath with magnetic stirring at 200°C for 2 h under nitrogen gas. The sediments were collected by centrifugation at 8,000 rpm, washed three times with a mixture of ethanol and n-hexane (1:1, v/v) and twice with ethanol, and ultimately re-dispersed in 1 mL of OAm; (2) 2 mg of Ni(acac)_2_, 3 mL of OAm, and 1 mL of an OAm solution of the product in step one were mixed in a 50-mL three-neck round-bottom flask and heated with magnetic stirring at 220°C for 2 h under the flow of nitrogen gas until the temperature decreased to room temperature. The precipitates were separated by centrifugation at 8,000 rpm, washed three times with a mixture of ethanol and n-hexane (1:1, v/v) and twice with ethanol, and eventually re-dispersed in 1 mL of OAm.

Synthesis of amorphous PdCuNiP nanoparticles. 1 mL of an OAm solution of Pd@PdCuNi core-shell nanocrystals, 1 mL of OAm, and 500 μL of TOP were mixed in a 50-mL three-neck round-bottom flask and heated under magnetic stirring at 290°C for 15 min under the flow of nitrogen gas until the temperature dropped to room temperature. The final product was initially centrifuged at 5,000 rpm followed by washing three times with a mixture of ethanol and n-hexane (1:1, v/v) and twice with ethanol.

### 2.3 Characterizations

Transmission electron microscopy (TEM) images were carried out on a Hitachi HT-7700 microscope equipped with a tungsten filament, operating at 100 kV. High-resolution TEM (HRTEM) imaging, high-angle annular dark-field scanning transmission microscopy (HAADF-STEM) imaging and energy-dispersive X-ray spectroscopy (EDS) elemental mapping were performed on a JEM-2100F (JEOL) equipped with a built-in EDS at 200 kV. The powder X-ray diffraction (XRD) patterns were recorded using an X-ray diffractometer (SmartLab (3), Rigaku) operated at 3 kW. X-ray photoelectron spectroscopy (XPS) was conducted using a Thermo Scientific K-Alpha spectrometer equipped with monochromatic Al Kα radiation. The contents of Pd, Cu, Ni, and P in the samples were received by inductively coupled plasma mass spectrometry (ICP-MS) with a PerkinElmer NexION 300X.

### 2.4 Electrocatalytic OER measurement

All the electrocatalysis tests were accomplished at 25°C in O_2_-saturated 1.0 M KOH electrolyte, using a typical three-electrode cell controlled by an electrochemical workstation (CHI 760E) with a catalyst-modified glassy carbon rotating disk electrode (RDE, diameter: 5 mm) as the working electrode, a platinum (Pt) foil as the counter electrode and a Hg/HgO (1.0 M KOH) as the reference electrode. It is worth noting that the electrochemical and chemical dissolution of a platinum foil counter electrode could take place in an alkaline electrolyte during catalysis, and cause redeposition of Pt on the working electrode, which has no obvious effect on the OER catalytic performance except for a small amount Pt dissolution and redeposition (Chen et al., 2017; Su et al., 2022). To prepare the working electrode, PdCuNiP, Pd@PdCuNi, Pd nanocubes, and commercial RuO_2_ catalysts were all deposited onto Ketjen Carbon (C) (EC300J) in ethanol with a noble metal loading of 20% (determined by ICP-MS). The products were separated by centrifugation and redispersed in a mixture of water, isopropanol, and 5 wt% Nafion (volume ratio, 1:1:0.02) under ultrasonication for 20 min to form a homogeneous ink. 10 µL catalyst ink was then loaded onto a precleaned RDE. All the potentials were calibrated in reference with the reversible hydrogen electrode (RHE) by the open circuit voltage test in 1 M KOH using the following equation:

$$E\left(vs.\;RHE\right)=E\left(vs.\;HgO\right)+0.904\;V$$ (1)

where 0.904 V is the potential difference between the Hg/HgO reference electrode and RHE in 1.0 M KOH. The OER activity was studied *via* linear sweep voltammetry (LSV) in the range of 1.3–1.8 V vs. RHE at 10 mV s^−1^ scan rate and 1,600 rpm rotation speed with 95% iR compensation. The overpotential (η) for OER could be calculated using the following equation:

$$\eta =E\left(vs.\;RHE\right)-1.23\;V$$ (2)

The electrochemical active surface area (ECSA) of the electrocatalysts was estimated from the double-layer capacitance (C_dl_). The C_dl_ depended on the cyclic voltammograms (CVs) measured in a non-faradaic potential region (0.9–1.0 V vs. RHE) in O_2_-saturated 1 M KOH at a series of different scan rates (10, 20, 30, 40, 50, and 60 mV s^−1^). And C_dl_ was calculated according to the following equation:

$$C_{dl}=\frac{∆J}{2v}$$ (3)

where ∆*J* is the current difference between the anode and cathode at 0.95 V vs. RHE, and v is the potential scan rate. Then, ECSA was obtained by the following equation:

$$ECSA=\frac{C_{dl}}{C_{s}}$$ (4)

where C_s_ is the specific capacitance of an atomically smooth planar surface [0.04 mF cm^−2^ in alkaline media (McCrory et al., 2013)]. Additionally, the roughness factor (R_f_) was estimated based on the equation below:

$$R_{f}=\frac{ECSA}{0.196\;{cm}^{2}}$$ (5)

where 0.196 cm^2^ is the geometric area of the electrode. Moreover, the specific current density (j_ECSA_) was normalized by ECSA value as the equation below:

$$j_{ECSA}=\frac{j_{Geo}}{R_{f}}$$ (6)

where j_Geo_ is the current density per geometric area of the electrode at a given overpotential. Electrochemical impedance spectroscopy (EIS) was tested at 1.5 V vs. RHE over a frequency range of 0.1–100K Hz with an amplitude of 5 mV at a rotation rate of 1,600 rpm. The catalytic stabilities were assessed by chronopotentiometry measurements at a current density of 10 mA cm^−2^ for 22 h.

## 3 Results and discussion

**FIGURE 1:**
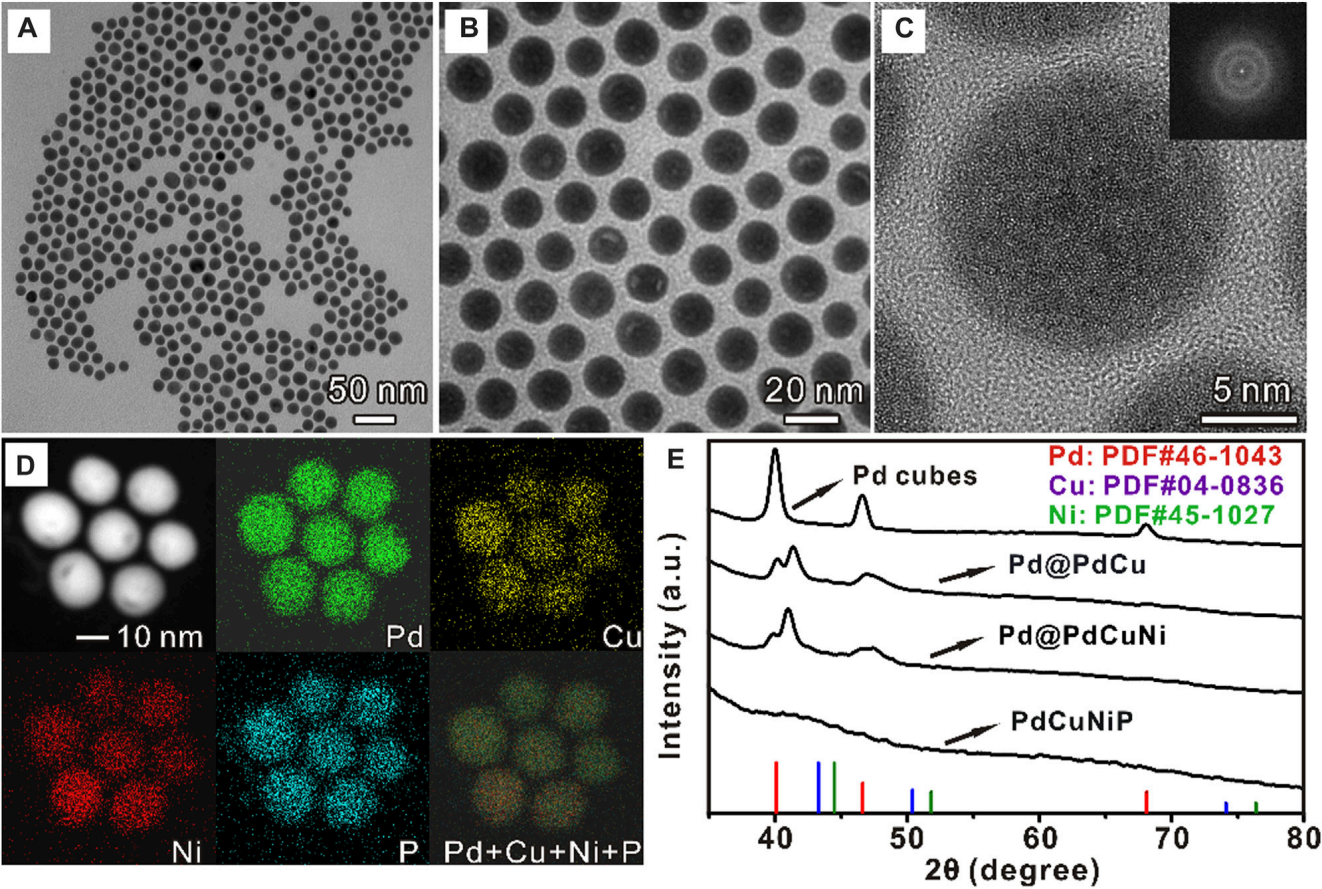
**(A, B)** TEM image, **(C)** HRTEM image, **(D)** EDS mappings of PdCuNiP nanoparticles, and **(E)** XRD patterns of Pd nanocubes, Pd@PdCu, Pd@PdCuNi, and PdCuNiP nanoparticles.

In a typical synthesis of PdCuNiP nanoparticles, Pd nanocubes were first prepared by the method reported previously (Jin et al., 2011). Supplementary Figure S1 shows a typical TEM image of the obtained Pd nanocubes, with sizes around 11 nm. Then, the ions of Cu and Ni were gradually added to the Pd solutions to reduce the second metals, and deposited onto the surface of Pd nanocubes, which subsequently diffused into the crystal lattices at the reaction temperature, finally resulting in the formation of Pd@PdCu and Pd@PdCuNi nanoparticles, respectively. Supplementary Figures S2, S3 show the TEM images of Pd@PdCu and Pd@PdCuNi nanoparticles, as well as the corresponding diagrams of the particle size distribution. As we can see, with the incorporation of Cu and Ni, the average size of the nanoparticles increases from 11 to 16 nm gradually, and the shape also slowly changes from cube to cuboctahedron. Then, the obtained Pd@PdCuNi nanoparticles were further subjected to the phosphorization treatment with TOP at 290°C. During the reaction, Phosphorus (P) atoms generated from the decomposition of TOP can insert into the lattice of nanoparticles, and thus the original crystalline core-shell nanoparticles would transform into amorphous spherical solid trimetallic PdCuNiP phosphide nanoparticles, as shown in Figure 1, Supplementary Figure S4. The average size of the PdCuNiP nanoparticles is ∼17 nm, slightly larger than that of the Pd@PdCuNi core-shell nanoparticles. Figure 1C reveals the HRTEM image of the PdCuNiP nanoparticles, where the amorphous structure of the products can be identified. Such an amorphous nature can also be evidenced by Fourier transformation (inset in Figure 1C). To further investigate the elemental composition and distribution, the Energy-dispersive X-ray spectroscopy (EDS) elemental mappings analysis was carried out (Figure 1D). PdCuNiP nanoparticles are composed of Pd, Cu, Ni, and P elements, while all the elements distribute homogeneously within the nanoparticles. The atomic ratio of Pd:Cu:Ni:P determined by inductively coupled plasma mass spectrometry (ICP-MS) is 44.2:16.2:25.2:14.4 for the amorphous trimetallic phosphide nanoparticles. Additionally, the chemical composition ratio of amorphous PdCuNiP nanoparticles can be further demonstrated by XPS test results listed in Supplementary Table S1, and these groups of atomic ratios are no different from each other. Figure 1E shows the XRD patterns of the Pd nanocubes, Pd@Cu, Pd@CuNi, and PdCuNiP nanoparticles. As can be seen in the XRD pattern, peak shifts could be observed for Pd@Cu and Pd@CuNi nanoparticles relative to the original Pd nanocubes (black line) due to the formation of alloy surfaces. Two peaks can be observed near 40° for Pd@PdCu and Pd@PdCuNi nanoparticles, which indicates the co-existence of Pd cores and shells and the formation of PdCu and PdCuNi alloy shells. However, the XRD pattern of PdCuNiP phosphide nanoparticles suggests that the Pd@PdCuNi core-shell nanocrystals have turned into an amorphous phase after phosphorization treatment, consistent with the HRTEM and Fourier transformation analysis. To further prove amorphous of PdCuNiP nanoparticles, the XRD pattern was presented with a wider 2*θ* range (Supplementary Figure S5).

**FIGURE 2:**
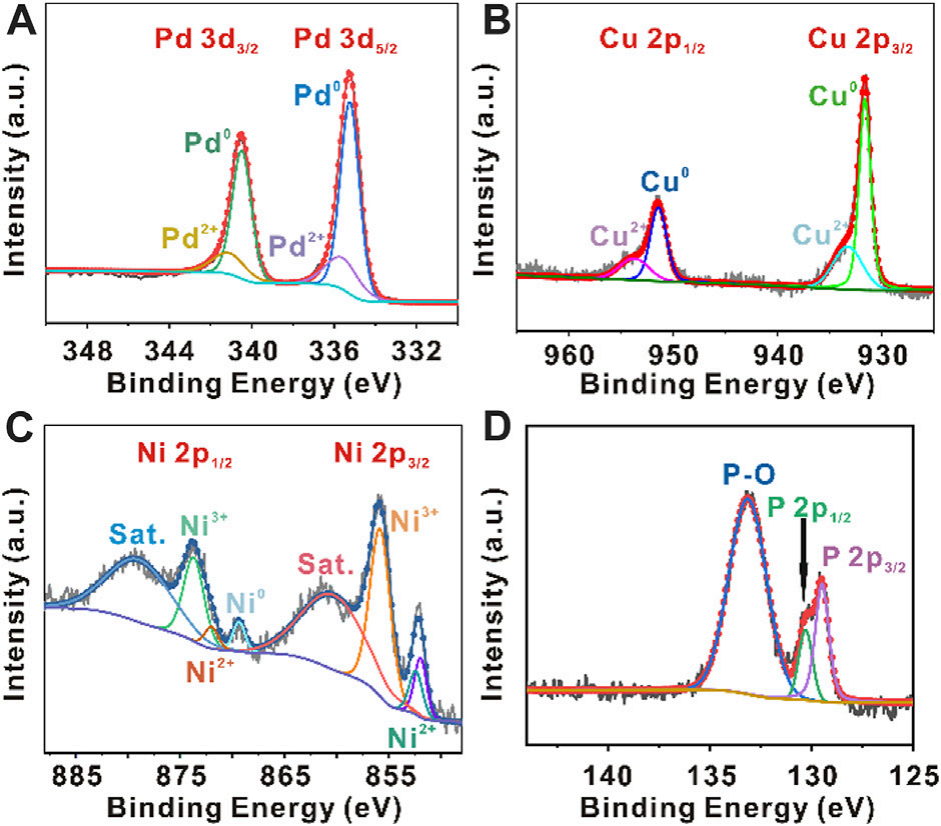
XPS survey spectra of sample PdCuNiP: **(A)** Pd 3d XPS spectra; **(B)** Cu 2p XPS spectra; **(C)** Ni 2p XPS spectra; and **(D)** P 2p XPS spectra.

XPS analysis was performed to investigate the chemical states and compositions of PdCuNiP nanoparticles (Figure 2). Figure 2A represents the Pd 3d_5/2_ and Pd 3d_3/2_ signals. As can be seen, the peaks can be deconvoluted into Pd^0^ and Pd^2+^ peaks. The peaks located at 335.2 and 340.5 eV could be attributed to metallic Pd (Pd^0^) and the peaks at 335.6 and 341.1 eV are from Pd^2+^. The peak intensity suggested that Pd^0^ is dominant in the product. Compared with the original Pd nanocubes, the Pd 3d_5/2_ and Pd 3d_3/2_ binding energy values of PdCuNiP nanoparticles shift by + 0.4 eV (Supplementary Figure S6). Figure 2B shows the high-resolution Cu 2p spectra. The binding energies 931.6 and 951.4 eV corresponds to zero-valance Cu, while the peaks at 933.2 and 951.4 eV correspond to Cu^2+^, respectively (Zhang et al., 2020). In the Ni 2p range, the XPS curves can be deconvoluted into three types of Ni species, including zero-valance Ni (Ni^0^), Ni^2+^, and Ni^3+^ (Figure 2C). In general, the binding energies at 852.0 and 869.4 eV could be attributed to Ni^0^, and the binding energies at 852.4, 855.8, 872.0, and 873.7 eV with shake-up satellite peaks (abbreviated as “Sat.”) at 860.3 and 878.9 eV correspond to the oxidized nickel species (Ni^2+^ and Ni^3+^), suggesting the existence of bi- and tri-valance nickel species on the surface of PdCuNiP nanoparticles (Hengne et al., 2018; Wang et al., 2019a; Jin et al., 2019; Lei et al., 2019; Qiu et al., 2019). In Figure 2D, the XPS P 2p spectra show two peaks at 129.5 and 130.3 eV corresponding to P 2p_3/2_ and P 2p_1/2_, respectively, which can be ascribed to the phosphide. This result further confirms the successful synthesis of trimetallic PdCuNiP phosphide nanoparticles in this work. The peak 133.1 eV assigned to P-O is mainly due to the inevitable surface binding of P with oxygen in the air. The coexisted Cu^2+^, Ni^2+^, Ni^3+^, and P-O could benefit the formation of oxygen-containing species (OH^−^_ads_) (Miao et al., 2014; Lv et al., 2019). According to the reports, this process can accelerate the reaction kinetics during OER, and thus improve the catalytic performance of the catalysts (Wang et al., 2017; Ibrahim et al., 2019). The increase of Pd, Cu, and Ni valence states is the result of electronic regulation between different elements, which is an important factor for improved OER performance. Also, valence states changes indicate the phenomenon of vast electron transfer from Pd, Cu, and Ni to P during the phosphorization treatment, contributing to the strong linkages between metal and P atoms, along with decreased 3d electron density and lower d-band energy of Pd, Cu, and Ni. Therefore, the electronic regulation weakened the bonding strength between the catalyst surface and the intermediate on the catalytic interface for enhanced OER catalytic performance (Zhou et al., 2006; Yang et al., 2010; He et al., 2021b). In addition, the positive shift of Pd 3d binding energies would also result in a stronger interaction with OH^−^_ads_, thereby enhancing the catalytic activity of Pd sites towards OER (Du et al., 2012; Bhowmik et al., 2016). Collectively, the above results reveal the chemical valence states and synergetic effects of Pd, Cu, Ni, and P elements in trimetallic amorphous phosphide nanoparticles.

**FIGURE 3:**
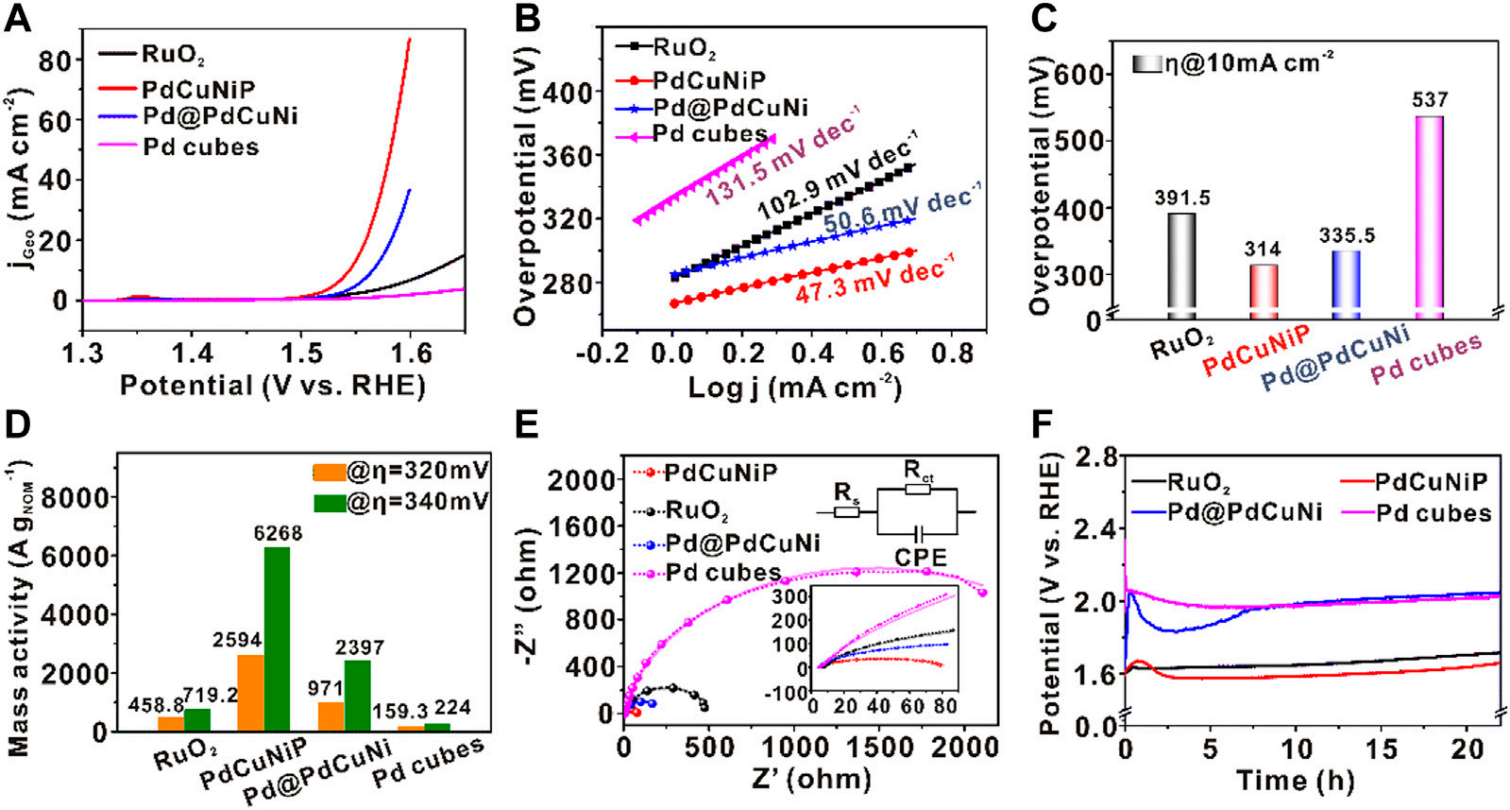
**(A)** LSV curves of PdCuNiP, Pd@PdCuNi, Pd nanocubes and commercial RuO_2_ for OER in 1 M KOH. **(B)** Corresponding Tafel plots derived from the data in **(A)**. **(C)** Comparison of the overpotentials among PdCuNiP, Pd@PdCuNi, Pd nanocubes, and commercial RuO_2_. **(D)** Mass activity of all catalysts at the overpotentials of 320 and 340 mV. **(E)** Nyquist plots of different catalysts. **(F)** Chronopotentiometry test of all catalysts for 22 h at the current densities of 10 mA cm^−2^.

The obtained trimetallic PdCuNiP amorphous nanoparticles were then evaluated as catalysts towards OER in 1.0 M O_2_-saturated KOH aqueous solution, benchmarking against the commercial RuO_2_, Pd@PdCuNi core-shell nanoparticles, and the original Pd nanocubes. Figure 3A shows the linear sweep voltammetry (LSV) normalized with the geometrical area of the electrode (0.196 cm^2^) at a scan rate of 5 mV s^−1^. The total mass of the noble metal loading was kept the same (5.1 μg cm^−2^) for all the catalysts. As shown in Figure 3A, in an alkaline medium, the activity sequence of the catalysts is PdCuNiP > Pd@PdCuNi > RuO_2_ > Pd nanocubes. The corresponding Tafel slopes of the catalysts are shown in Figure 3B. Different from the original Pd nanocubes (131.5 mV dec^−1^), RuO_2_ (102.9 mV dec^−1^), and Pd@PdCuNi (50.6 mV dec^−1^), the Tafel slope of PdCuNiP sharply decreased to 47.3 mV dec^−1^, which should be ascribed to the synergistic of trimetallic component, the modified electronic structure, and the amorphous structure of the catalyst, which helps to lower the energy barrier and thus accelerate the reaction kinetics. Figure 3C further shows that the prepared trimetallic PdCuNiP phosphide nanoparticles possess the lowest overpotential (314 mV) to afford a current density of 10 mA cm^−2^ in OER, which is 223, 77.5, and 21.5 mV lower than those of the original Pd nanocubes, commercial RuO_2_ catalysts, and the Pd@PdCuNi core-shell catalysts, respectively. The above experiment results manifest that the synergistic effect between the trimetallic component and the amorphous structure, as well as the introduction of P, can significantly enhance the electrocatalytic performance. Moreover, the mass activities of the catalysts normalized to noble metal loadings (measured by ICP-MS, Supplementary Figure S7), which is a vital indicator of electrocatalytic activity in practical applications (Zhao et al., 2017), were further calculated based on the values measured at the overpotentials of 320 and 340 mV (Figure 3D). As can be seen, the mass activity of PdCuNiP achieves 2,594 and 6268 A g^−1^_Pd_ at overpotentials of 320 and 340 mV, respectively, which is 5.7 and 8.7 times as high as that of the commercial RuO_2_ catalyst. The electrochemical reaction kinetics were further investigated using electrochemical impedance spectroscopy (EIS). Figure 3E illustrates the Nyquist plots fitting the equivalent circuit diagram at the potential of 1.50 V vs. RHE for different electrocatalysts, in which solid lines represent experimental data and dotted lines with circles represent fitting curves. The obtained solution resistance (R_s_) is almost equivalent for all the catalysts, and the diameters of the fitting semicircles accord with the charge transfer resistance (R_ct_). Compared with other samples, the R_ct_ of PdCuNiP is much lower, suggesting a faster transport rate of electrons and reaction kinetics towards oxygen evolution reaction (Supplementary Table S2). Unambiguously, the results further confirm the enhanced intrinsic activity for amorphous PdCuNiP nanoparticles. The stability of the catalysts is another important parameter. As shown in Figure 3F, the chronoamperometry measurements were employed to test the stability of the catalysts. After testing for 22 h, the overpotential decrease of PdCuNiP is 66 mV, which is much smaller than those of Pd@PdCuNi nanoparticles (412 mV), Pd cubes (84 mV), and commercial RuO_2_ (180 mV). The reason that the activity of PdCuNiP catalysts decreased first and then increased significantly in Figure 3F is caused by the oxidation of the oleylamine and other organic compounds adsorbed by the catalysts during the stability test. In addition, it was found that the PdCuNiP catalyst did not undergo structural reconstruction by testing the HRTEM and XRD of the catalyst after the stability test, and the amorphous structure is well maintained (Supplementary Figure S9). This is different from the previously reported surface reconstruction process of some metal phosphides and metal-based compounds (PdCuNiP, cobalt pnictide, and defect-rich Co_3_O_4_) during OER (Kim et al., 2018; Lyu et al., 2020; Huang et al., 2022). Worthy, the PdCuNiP catalyst exhibits outstanding performance compared to other reported catalysts listed in Supplementary Table S3 for OER (Bai et al., 2016; Guan et al., 2017; He et al., 2017; Liyanage et al., 2017; Ren et al., 2017; Wang et al., 2019b).

**FIGURE 4:**
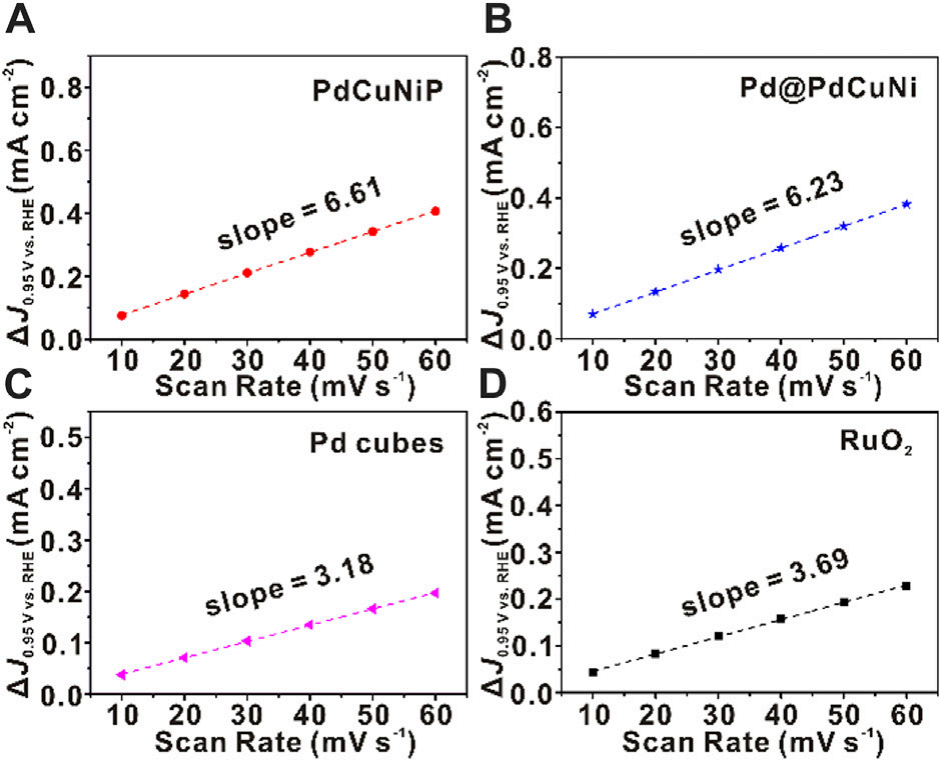
Measurements for electrochemical double-layer capacitance. Corresponding ∆*J* as a function of scan rates of **(A)** PdCuNiP, **(B)** Pd@PdCuNi, **(C)** Pd nanocubes and **(D)** RuO_2_ catalysts.

To better illustrate the intrinsic activity of the catalysts towards OER, the electrochemical activity surface area (ECSA) and roughness factor (R_f_) were calculated by measuring the electrochemical double-layer capacitance (C_dl_) of corresponding electrocatalysts. As shown in Supplementary Figure S10, cyclic voltammetry (CV) curves were carried out at a fixed potential (0.90–1.0 V vs. RHE) from the non-faradaic potential with various scan rates from 10 to 60 mV s^−1^. As evidenced in Figure 4, the curves of the difference between anodic and cathodic current densities (∆*J*) against scan rate were plotted, in which the fitting slops represent twice of C_dl_. The calculated results summarized in Supplementary Table S4 show that the C_dl_ and ECSA of PdCuNiP nanoparticles were 3.31 mF cm^−2^ and 82.75 cm^2^ g^−1^_Pd_, respectively, which were superior to that of Pd@PdCuNi (3.12 mF cm^−2^ and 78 cm^2^ g^−1^_Pd_), Pd nanocubes (1.59 mF cm^−2^ and 39.75 cm^2^ g^−1^_Pd_) and RuO_2_ (1.85 mF cm^−2^ and 46.25 cm^2^ g^−1^_Ru_). It turns out that PdCuNiP nanoparticles would possess more active sites, which could be attributed to the low coordination surface of amorphous structure (Tsuji et al., 2011; Bergmann et al., 2015; Zhao et al., 2017; Liu et al., 2018b; Anantharaj et al., 2020). Additionally, to reduce the impact of the different ECSAs, we further compared the intrinsic activity by recording the ECSAs normalized polarization curves and a diagram of the specific activity versus different overpotentials of 320 and 340 mV for all the samples (Supplementary Figure S11; Supplementary Table S4). As can be seen, the trends of the C_dl_, ECSA, and R_f_ were found to be consistent with that of the activity. As well, the computed C_dl_ increased with the growth of the particle size, which agreed with our theoretical predictions, and means the trend of the C_dl_, ECSA and R_f_ coincide with that of MA, SA, and the particle sizes (Supplementary Table S4; Supplementary Figures S2–S4). Collectively, the prepared trimetallic amorphous PdCuNiP nanoparticles exhibit the largest ECSA, and the highest specific and mass activities among the four catalysts, showing the important role of the synergistic effect of multi-elements and the amorphous structure in improving the catalytic performance of noble-metal based electrocatalysts.

## 4 Conclusion

In summary, amorphous structured trimetallic PdCuNiP nanoparticles with enhanced OER performance under an alkaline solution have been synthesized through alloying Cu and Ni into the surface lattice of Pd nanocubes and the subsequent phosphorization treatment. Catalytic performance evaluation reveals that both the synergistic effect between four kinds of elements and the advantage of amorphous structure can enhance the catalytic performance of the Pd-based catalysts. Hence, trimetallic amorphous PdCuNiP phosphide nanoparticles can show a mass activity nearly 20-fold enhancement compared with the initial Pd nanocubes towards OER, and an overpotential 223 mV lower. This work may shed new light on both the fabrication of novel amorphous multi-metallic phosphide electrocatalysts and their catalytic applications in a set of electrocatalysis, thereby promoting the practical applications of electrocatalysis in renewable energy conversion systems.

## Data Availability

The original contributions presented in the study are included in the article/Supplementary Material, further inquiries can be directed to the corresponding authors.
