# Supplementary material for: Synthesis of amorphous trimetallic PdCuNiP nanoparticles for enhanced OER
Source: Front Chem. 2023 Jan 30;11:1122333. doi: 10.3389/fchem.2023.1122333 (PMC9922906; doi:10.3389/fchem.2023.1122333)
Supplement: Supplementary file 1 [file DataSheet1.PDF]

Supplementary Material

Synthesis of amorphous trimetallic PdCuNiP nanoparticles for enhanced OER

Yangzi Zheng[1], Ruiyun Guo[2,*], Xiang Li[3], Tianou He[1], Weicong Wang[1], Qi Zhan[1], Rui Li[1], Ke Zhang[1], Shangdong Ji[1], Mingshang Jin[1,*]

[1]Frontier Institute of Science and Technology and State Key Laboratory of Multiphase Flow in Power Engineering, Xi’an Jiaotong University, Xi’an, Shaanxi 710049, China

[2]School of Materials Science and Engineering, Xi’an University of Science and Technology, Xi’an, Shaanxi 710054, China

[3]Shaanxi Key Laboratory of Optoelectronic Functional Materials and Devices, School of Materials Science and Chemical Engineering, Xi'an Technological University, Xi'an, Shaanxi 710021, China.

*** Correspondence:**
Mingshang Jin, Email: jinm@mail.xjtu.edu.cn

Ruiyun Guo, Email: guoruiyun@stu.xjtu.edu.cn

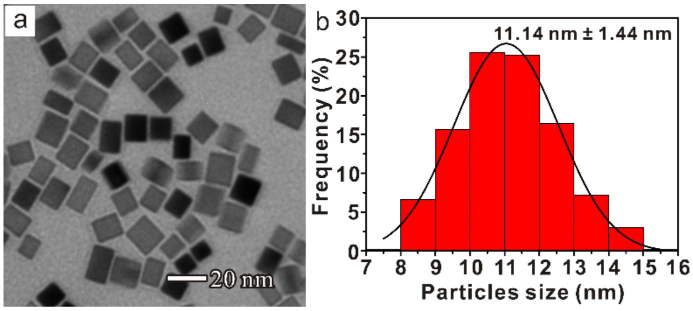

Supplementary Figure 1. a) TEM image of Pd nanocubes and b) the corresponding diagram of the particle size distribution.

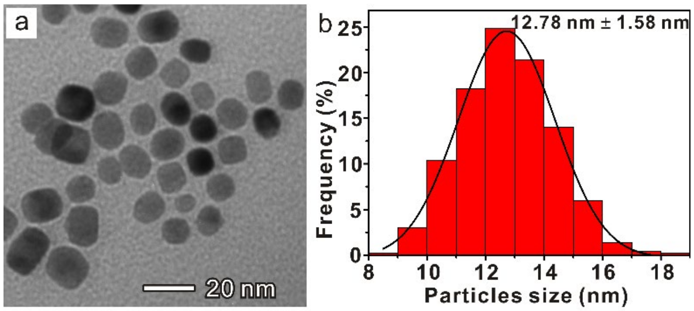

Supplementary Figure 2. a) TEM image of Pd@PdCu nanoparticles and b) the corresponding diagram of the particle size distribution.

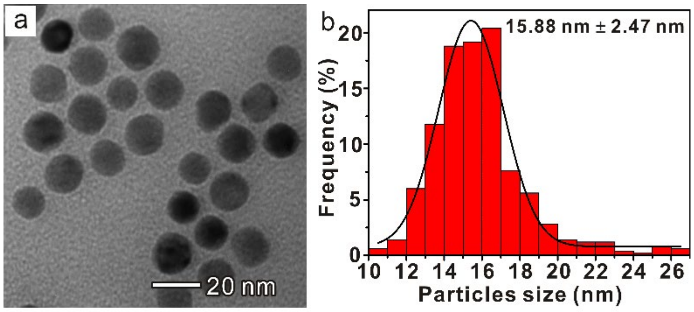

Supplementary Figure 3. a) TEM image of Pd@PdCuNi nanoparticles and b) the corresponding diagram of the particle size distribution.

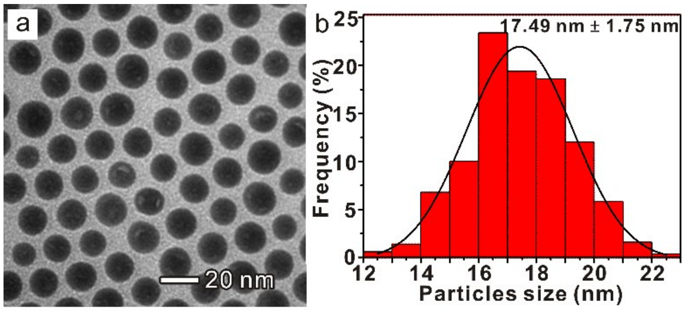

Supplementary Figure 4. a) TEM image of PdCuNiP nanoparticles and b) the corresponding diagram of the particle size distribution.

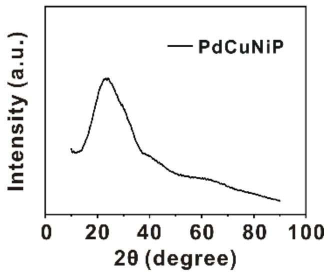

Supplementary Figure 5. XRD pattern of PdCuNiP nanoparticles.

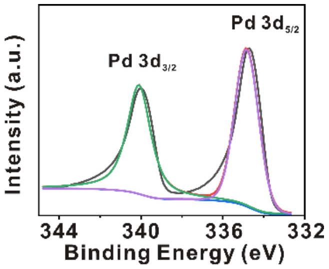

Supplementary Figure 6. XPS survey spectra of Pd nanocubes.

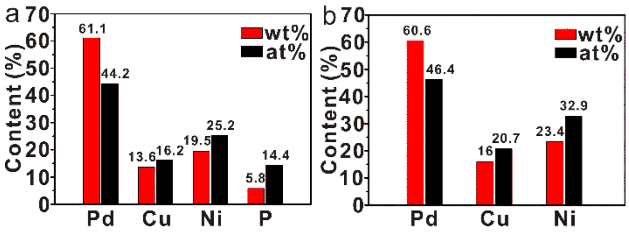

Supplementary Figure 7. ICP-MS analyses of prepared a) PdCuNiP and b) Pd@PdCuNi nanoparticles.

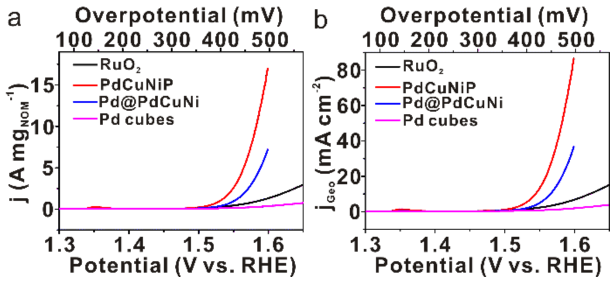

Supplementary Figure 8. a) Mass activities and b) LSV curves of PdCuNiP, Pd@PdCuNi, Pd nanocubes, and commercial RuO_2 for OER in 1 M KOH.

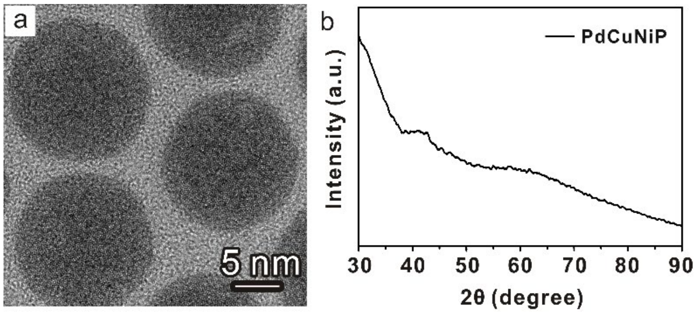

Supplementary Figure 9. a) The HRTEM image and b) XRD pattern of the PdCuNiP catalyst after the stability test.

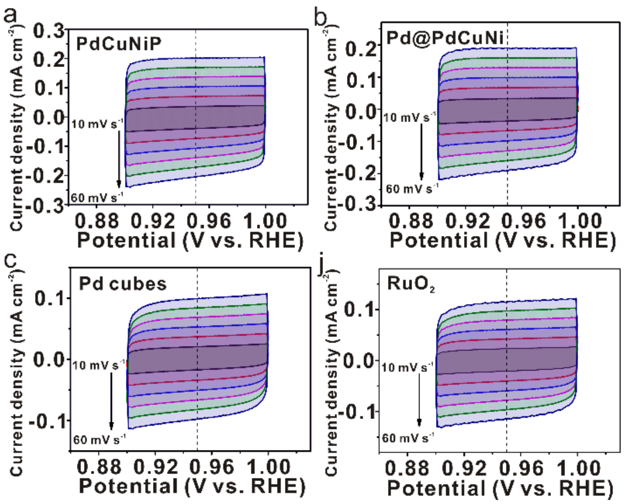

Supplementary Figure 10. CV curves at different scan rates from 10 to 60 mV s^{-1} of a) PdCuNiP, b) Pd@PdCuNi, c) Pd nanocubes and d) RuO_2.

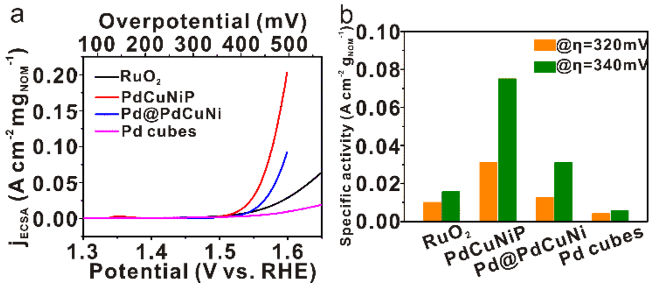

Supplementary Figure 11. a) ECSA normalized polarization curves and b) specific activity at the overpotential of 320 and 340 mV of PdCuNiP, Pd@PdCuNi, Pd nanocubes, and commercial RuO_2 for OER in 1 M KOH.

Supplementary Table 1. The chemical composition of amorphous PdCuNiP nanoparticles obtained by XPS test.

Elements	Pd	Cu	Ni	P
Atomic ratio	42.3	16.6	25.2	15.9

Supplementary Table 2. Comparison of the R_s and R_{ct} among PdCuNiP, Pd@PdCuNi, commercial RuO_2, and Pd nanocubes.

Catalysts	PdCuNiP	Pd@PdCuNi	RuO_2	Pd nanocubes
R_s (ohm)	4.9	4.7	7.1	4.8
R_{ct} (ohm)	80.6	220.4	464.6	3005.2

Supplementary Table 3. Comparison of the catalytic activity toward the OER between PdCuNiP nanoparticles and other reported catalysts.

Catalysts	Overpotential (at 10 mA cm^{-2})	Tafel slope (mV dec^{-1})	Refers
Amorphous PdCuNiP nanoparticles	314	47.3	This work
Mn-Co oxyphosphide	320	52	
$Ni_{2-x}Ru_xP$ particles	340	/	
Few-layer BP	>400	75	
NiCoP/C nanoboxes	330	96	
Co-P/Co-N-C/NPC	370	92	
CoP/C	330	53	

Supplementary Table 4. Comparison of the calculated C_{dl}, ECSA, R_f, MA, and SA among PdCuNiP, Pd@PdCuNi, commercial RuO_2, and Pd nanocubes.

Catalysts	C_{dl} (mF cm^{-2})	ECSA (cm^2 g^{-1}_{NOM})	R_f	MA (A g^{-1}_{NOM}) at η = 320mV	SA (A cm^{-2} g^{-1}_{NOM}) at η = 320 mV
PdCuNiP	3.31	82.75	422.19	2594	31.03
Pd@PdCuNi	3.12	78	397.96	971	12.47
RuO_2	1.85	46.25	235.97	458.8	9.95
Pd cubes	1.59	39.75	202.81	159.3	4.01
